# The inflammatory and normal transcriptome of mouse bladder detrusor and mucosa

**DOI:** 10.1186/1472-6793-6-1

**Published:** 2006-01-18

**Authors:** Marcia R Saban, Helen L Hellmich, Mary Turner, Ngoc-Bich Nguyen, Rajanikanth Vadigepalli, David W Dyer, Robert E Hurst, Michael Centola, Ricardo Saban

**Affiliations:** 1 Department of Physiology, The University Oklahoma Health Sciences Center, Oklahoma City, USA; 2 Department of Anesthesiology, University of Texas Medical Branch, Galveston, USA; 3 Oklahoma Medical Research Foundation (OMRF), Arthritis and Immunology Research Program, Microarray Core Facility, Oklahoma City, USA; 4 Daniel Baugh Institute for Functional Genomics and Computational Biology. Department of Pathology, Anatomy and Cell Biology, Thomas Jefferson University, Philadelphia, USA; 5 Department of Microbiology and Immunology, Laboratory for Genomics and Bioinformatics, Oklahoma University Health Sciences Center, Oklahoma City, USA; 6 Department of Urology, The University Oklahoma Health Sciences Center, Oklahoma City, USA; 7 Cellular & Structural Biology, The University of Texas Health Science Center at San Antonio, San Antonio, USA

## Abstract

**Background:**

An organ such as the bladder consists of complex, interacting set of tissues and cells. Inflammation has been implicated in every major disease of the bladder, including cancer, interstitial cystitis, and infection. However, scanty is the information about individual detrusor and urothelium transcriptomes in response to inflammation. Here, we used suppression subtractive hybridizations (SSH) to determine bladder tissue- and disease-specific genes and transcriptional regulatory elements (TRE)s. Unique TREs and genes were assembled into putative networks.

**Results:**

It was found that the control bladder mucosa presented regulatory elements driving genes such as myosin light chain phosphatase and calponin 1 that influence the smooth muscle phenotype. In the control detrusor network the Pax-3 TRE was significantly over-represented. During development, the Pax-3 transcription factor (TF) maintains progenitor cells in an undifferentiated state whereas, during inflammation, Pax-3 was suppressed and genes involved in neuronal development (*synapsin I*) were up-regulated. Therefore, during inflammation, an increased maturation of neural progenitor cells in the muscle may underlie detrusor instability. NF-κB was specifically over-represented in the inflamed mucosa regulatory network. When the inflamed detrusor was compared to control, two major pathways were found, one encoding *synapsin I*, a neuron-specific phosphoprotein, and the other an important apoptotic protein, *siva*. In response to LPS-induced inflammation, the liver X receptor was over-represented in both mucosa and detrusor regulatory networks confirming a role for this nuclear receptor in LPS-induced gene expression.

**Conclusion:**

A new approach for understanding bladder muscle-urothelium interaction was developed by assembling SSH, real time PCR, and TRE analysis results into regulatory networks. Interestingly, some of the TREs and their downstream transcripts originally involved in organogenesis and oncogenesis were also activated during inflammation. The latter represents an additional link between inflammation and cancer. The regulatory networks represent key targets for development of novel drugs targeting bladder diseases.

## Background

The lower urinary tract is subject to a number of functional disorders in which a cross-communication between urothelium and detrusor muscle is a factor. Bladder overactivity has been attributed to detrusor muscle dysfunction, and several *in vitro *and *in vivo *methodologies have been developed to better understand its pathophysiology [1]. Although the detrusor muscle participates intensively in the inflammatory response, practically every major disease of the urinary bladder, including cancer, interstitial cystitis, and infection [2], involves the urothelium [3].

The urinary bladder develops as a result of indispensable epithelial-mesenchymal interactions responsible for directing urothelial differentiation and for normal smooth muscle development [4,5]. However, in adulthood, the urinary bladder is a highly heterogeneous organ, consisting of a large variety of cell types, and this complexity presents challenges to the study of physiological and cellular processes in health and disease. This was evident in studies determining gene regulation [6-11] and target validation [12]. In the latter study, the expression of protease-activating receptors (PAR) was differentially distributed between bladder mucosa, detrusor smooth muscle, and nerve elements. Moreover, during inflammation, PAR expression was up-regulated in the mucosa contrasting with its down-regulation in the detrusor muscle [12]. These results suggested a possible differential distribution of proteins between bladder mucosa and detrusor muscle and indicated the need for reduction of the whole tissue into specific layers.

The present work was undertaken to elucidate the transcriptional complexity of inflammation, as modeled in the mouse urinary bladder. The first step towards the study of individual layers was the separation of the mucosa and submucosa layers away from the detrusor smooth muscle and adventitia (See Additional file 1). We went further to determine low abundant transcripts, using suppression subtractive hybridization (SSH) and selected ones were confirmed by real time PCR. Next, the SSH-originated transcripts were annotated and analyzed for significantly enriched transcriptional regulatory elements (TRE) using PAINT [Promoter Analysis and Interaction Network Toolset; [13]]. PAINT utilizes TRANSFAC database [14] containing eukaryotic cis-acting regulatory DNA elements and trans-acting factors. The pattern search tool MATCH in TRANSFAC suite is employed to identify the TREs on cognate 5' upstream regulatory sequences [15]. Putative regulatory networks were assembled using known interactions among genes and their coded proteins as well as information about TREs that were significantly over-represented in the genes comprising inflammatory and control transcriptomes. Together, these results constitute the first demonstration of the transcriptional complexity underlying the different layers of the urinary bladder and their contribution to the early phases of bladder inflammation.

## Results

*SSH*. From each library, at least 50 clones were further sequenced and annotated, for a total of 300 clones. The present work reports only tissue-specific transcripts (mucosa vs detrusor) and treatment-specific transcripts (saline vs LPS). 120 transcripts were considered to be unique. These indicate that each of these 120 transcript occurred in a specific subtraction. Transcripts that appeared in more than one subtraction were not considered unique and therefore, not included. Selection of clones ceased when a substantial number of additional sequences [approximately 180] did not reveal any additional unique transcript. Next, 120 clones were sequenced and analyzed for homology in the GenBank and EMBL databases.

Seventy six cDNA fragments (Table 2) were further annotated using the Mouse Genome Information [16] according to Gene Ontology and presented in Table 3. Among the 76 fragments selected for futher annotation, 21 fragments had homology with expressed sequence tags (ESTs) and cDNA clones for which information about tissue specificity, biologycal or molecular function is not available. Interestingly, one of these clones (*ambladder*; RIKEN clone:9530014P05) was originally isolated from an adult male bladder cDNA library [17].

**Table 1:** Primers for real time PCR

***Gene***	***GenBank ID***	***5' primer sequence***	***3' primer sequence***	***localization***
Bcap31	NM_009922	AAAGAATATGACCGCCTGCTAGA	AAGCCTTTACTCCTCCTTCTTGACT	761–847
Catenin	BC043108	CCTCCCCCACCCACATCTA	ACCCACACCCCACCGAgaa	4957–5049
Cnn1	NM_009922	AGTTGTTTGCTGCCAAGTCTGA	GGTGGAAGGCAGTTTAATGGAGT	2081–2168
Dlk1	D16847	CTTTTTGTGGTGGAGTTTGCTCTAT	gCGTGGTAGCATGGCACACA	1859–1950
eiF-4E	NM_010124	TGTGCTTGGCTGCTGAGAGA	ACGGACAGACGGACGATGA	1446–1531
Grn	X62321	CTACCTAAAGGGTGTCTGCTGTAGA	AGGAATCTTCTTTCGCAAACACTT	1630–1729
Grp58	BC003285	AAAACCAGAGAGGACAGAATGGATAA	TGTATTTTCAAACAGTGCAGCTAAGAA	1627–1712
GSTM1	NM_010358	TCTCCTTCCCGCTCCCTT	GAGAATGAAGGCTGTGTGGACTT	965–1047
GSTO1	NM_010362	GGCAAGAGCCCTCAGCAA	TGAGAAAGGAGCCAGTGAGAATACT	826–912
IFIT3	BC003804	TGTGGTGGATTCTTGGCAGTT	CTGCCTGTGCCCCAAAGT	1279–1366
LAMP2	NM_010685	TGGCACTGGCTTAATGCTGTT	GTGCTTTGGAGGTATCTCAATATGAA	3132–3218
Mafb	AF180338	CCATCTTGAGAAGGTAGCAGCAA	AAAGTTGGGCTTGGTGGGTT	3731–3840
Mpp1	U38196	AGATTGCCATCCTTGACATTGA	GTAGGTGCGATGAACACAATGAA	1301–1388
Pls3	NM_145629	CACACCCAGGCTCAAAGGA	TTGTGATAAAGATTTCCAAACAAACAA	2816–2902
Prss11	NM_019564	AGTCAACATTTGTCCCTTCCCTTA	GGCTGCGAGGACCTTCCT	1752–1837
Sirt1	AF214646	CCTGCATAGATCTTCACCACAaat	ACACTCTCCCCAGTAGAAGTACCATT	3016–3108
Smoc2	NM_022315	CCACTATGGGATGAAGGTTATGA	AGAAAGTGACAGCCAGCCATACA	2377–2465
SPRR2A	BC010818	ATAGCAACACTTCCATCCTCCTTT	TGAGGAGCCATCATAAGCACAT	379–471
SYN1	NM_013680	GTTCTAAAGTCATCGTTCGGTTCTTAA	TTCCCAGCTCTGTGATCATCAA	3334–3424
TMPRSS2	AF199362	AATCACACCAGCCATGATCTGT	AATCAGCCACCAGATCCCATT	1364–1473
Upk1a	AF262335	GGCAACTTCATCCCCATCAA	AGCAACCCTTGGTAAACAGGTAGT	580–652

**Table 2:** Mouse bladder transcripts isolated by SSHs

*Abbrev*	*Name*	*Accession*	*Library*	*QPCR*
2310015N07	RIKEN cDNA 2310015N07 gene	NM_025515	MIDI	N
Actg1	actin, gamma 1, cytoplasmic	NM_009609	MIC	N
Actg2	actin, gamma 2, smooth muscle, enteric	NM_009610	MIDI	N
Actr3	ARP3 actin-related protein 3 homolog (yeast)	NM_023735	MIC	N
Ambladder	adult male urinary bladder cDNA, RIKEN clone:9530014P05	AK020558	MC	N
Amcq	adult male corpora quadrigemina cDNA, RIKEN clone:B230340L02	AK080832	MIC	Y
Aplp2	amyloid beta (A4) precursor-like protein 2 isoform 751	U15571	MIDI	N
Bcap31	B-cell receptor-associated protein 31	NM_012060	MIDI	Y
Calm2	calmodulin 2	NM_007589	MC	N
Catenin	similar to catenin src	BC043108	MIC	N
Cnn1	calponin 1	NM_009922	MC	N
Col3a1	collagen, type III, alpha 1, RIKEN 3200002K15	AK019448	MIDI	N
Cox7b	cytochrome c oxidase subunit VIIb	NM_025379	MIC	N
CoxI	mitochondrial gene for subunit I of cytochrome c oxidase	X57780	MIC	N
Cpe	carboxypeptidase E (Cpe),	NM_013494	MIC	N
Cts e	cathepsin E (Ctse)	NM_007799	MIC	N
Cts h	cathepsin H	NM_007801	DC	N
Cts l	cathepsin L	NM_009984	MIC	Y
Ddx3	DEAD/H (Asp-Glu-Ala-Asp/His) box polypeptide 3	NM_010028	MIDI	Y
DnaJ	Hsp40 homolog, subfamily A, member 2	NM_019794	MIDI	N
Eif4ebp2	eukaryotic translation initiation factor 4E binding protein 2	NM_010124	DIC	N
Elp3	elongation protein 3 homolog, RIKEN clone:2610507P14	AK012072	MIDI	Y
Endomuc1	endomucin-1	BC003706	MIC	N
Fth1	ferritin heavy chain 1	NM_010239	MIC	N
Gpam/GPAT	glycerol-3-phosphate acyltransferase	NM_008149	MIC	Y
Grn	epithelin 1 and 2 (granulin)	X62321	MC	N
Grp58	glucose regulated protein	BC003285	MIC	Y
Gst a4	glutathione S-transferase, alpha 4	NM_010357	MIDI	N
Gst m1	glutathione S-transferase, mu 1	NM_010358	DC	Y
Gst o1	glutathione S-transferase omega 1	NM_010362	DC	Y
Gus	beta-glucuronidase	NM_010368	MIDI	N
IFIT3	interferon-induced protein with tetratricopeptide repeats 3	BC003804	MIC	Y
Ifrg15	interferon alpha responsive gene	NM_022329	DC	N
Lamp2	lysosomal membrane glycoprotein 2	NM_010685	MIC	N
Lrrfip2	leucine rich repeat (in FLII) interacting protein 2	XM_284541	MIDI	Y
Ly6d	mRNA for THB / lymphocyte antigen 6 complex, locus D	X63782	MIDI	N
Mafb	transcription factor MAFB	AF180338	MIC	N
Mgs1-182e11	clone mgs1-182e11 strain 129/SvJ	AC096622	MIDI	Y
MLZE	adult female vagina cDNA, RIKEN clone 9930109F21	AK037079	MIDI	N
Mpp1	palmytoylated protein p55	U38196	DIC	N
My h11	myosin heavy chain 11, smooth muscle	XM_147228	MIDI	Y
My lc2b/MRLC2	myosin light chain, regulatory B	NM_023402	MC	Y
My19	myosin, light polypeptide 9, regulatory	XM_283793	MIDI	N
Pls3	similar to plastin 3 precursor (T-isoform)	NM_145629	MIC	Y
Prdx1	peroxiredoxin 1	NM_011034	MIDI	N
Prnp	prion protein	NM_011170	DC	Y
Prss11	protease, serine, 11 (Igf binding) HtrA1	NM_019564	MIC	N
RP23-452N23	clone RP23-452N23 on chromosome 4	AL928645	MIC	N
RP23-70M6	chromosome 18 clone RP23-70M6	AC114820	MIC	N
RPCI23	Strain C57BL6/J Chromosome 2, clone RP23-111A22	AC078911	MIDI	Y
RPs21	ribosomal protein S21 / RIKEN cDNA 2410030A14 gene	BC027563	MIC	N
RPs7	ribosomal protein S7	NM_011300	MIDI	Y
SAC1	suppressor of actin mutations	AJ245720	MC	Y
SC1	extracellular matrix protein precursor	U77330	MC	N
SCP-1	SCP-1 mRNA for stromal cell derived protein-1	D16847	MIC	Y
Sdfr1	stromal cell derived factor receptor 1	NM_009145	DC	N
Sepp1/SePP	selenoprotein P, plasma,1 glycoprotein	NM_009155	MIC	Y
Serpina3n	serine (or cysteine) proteinase inhibitor (clade A, member 3N)	NM_009252	DC	Y
Sf 3b2	splicing factor 3b, subunit 2, clone MGC:61326 IMAGE:6812422	BC049118	MIDI	N
Sf rs6	splicing factor, arginine/serine-rich 6	NM_026499	MIC	N
Sf sc35	splicing factor SC35	AF077858	MIDI	N
Sirt1	Sir1 alpha protein	AF214646	MIDI	N
Siva	Cd27 binding proapoptotic protein	NM_013929	DIC	Y
Smoc2	SPARC related modular calcium binding 2	NM_022315	MIC	N
Sparc	secreted acidic cysteine rich glycoprotein	NM_009242	MIDI	N
Sprr 2A	small proline-rich protein 2A	BC010818	MC	Y
Syn1	synapsin I or ribosomal protein S15a	NM_013680	DIC	Y
Tcra-V13.1	T-cell receptor alpha/delta locus	AE008684	MIC	N
Thsd6	thrombospondin, type I domain containing 6	NM_025629	DC	Y
TMPRSS2	transmembrane protease, serine 2	NM_015775	MIDI	N
Tnnt3	troponin T3, skeletal, fast	NM_011620	MIDI	N
UDP-gluco	UDP-glucuronosyltransferase 1 family, member 2	BC019434	MIC	N
Upk1a	uroplakin 1a	AF262335	DC	N
Upk1b	uroplakin 1b	NM_178924	DC	N
Wdr1	WD repeat domain 1	NM_011715	MIDI	N
Zfp364	zinc finger protein 364/Rab7	NM_026406	MIDI	N

**Table 3:** ANNOTATION (GENE ONTOLOGY) OF SSH-ISOLATED TRANSCRIPT FROM MOUSE URINARY BLADDER

*Biological Process*	*Abbrev*	*Molecular Function*	*Cellular Component*	*Accession*
actin dynamics	Wdr1	actin binding	actin cytoskeleton	NM_011715
apoptosis	Sirt1	NAD-dependent histone deacetylase	chromatin silencing complex	AF214646
apoptosis	Bcap31	receptor binding	Golgi membrane	NM_012060
apoptosis	Siva	CD27 receptor binding	cytoplasm	NM_013929
calcium ion binding	SCP-1	calcium ion binding	integral to membrane	D16847
calcium ion binding	Sparc	calcium ion binding	basement membrane	NM_009242
calcium ion binding	Smoc2	calcium ion binding	extracellular space	NM_022315
calcium ion binding	Pls3	calcium ion binding	unknown	NM_145629
calcium ion binding	SC1	calcium ion binding	extracellular space	U77330
cell adhesion	Col3a1	extracellular matrix structural constituent	collagen	AK019448
cell adhesion	Catenin	protein binding	cytoskeleton	BC043108
cell cycle/G-protein coupled receptor	Calm2	protein binding	plasma membrane	NM_007589
cell growth (regulation)	Prss11	insulin-like growth factor binding	extracellular region	NM_019564
cell motility	Actr3	structural molecule	actin cytoskeleton	NM_023735
cytoskeleton organization and biogenesis	Actg1	motor activity	actin cytoskeleton	NM_009609
cytoskeleton organization and biogenesis	Actg2	motor activity	actin cytoskeleton	NM_009610
cytoskeleton organization and biogenesis	My lc2b	unknown	cytoskeleton	NM_023402
defense response	Ly6d	unknown	plasma membrane	X63782
electron transport	Grp58	electron transporter	endoplasmic reticulum	BC003285
electron transport	Cox7b	oxidoreductase activity	mitochondrial electron transport chain	NM_025379
electron transport	CoxI	ubiquinol-cytochrome-c reductase activity	mitochondrion	X57780
epithelial cell proliferation	Grn	phospholipase A2	mitochondrion	X62321
immune response	IFIT3	unknown	unknown	BC003804
insulin processing	Cpe	carboxypeptidase A and E activity	extracellular space	NM_013494
iron ion transport	Fth1	ferric iron binding	unknown	NM_010239
metabolism	Gst a4	glutathione transferase	unknown	NM_010357
metabolism	Gst m1	glutathione transferase	unknown	NM_010358
metabolism	Gst o1	glutathione transferase	cytoplasm	NM_010362
negative regulation of translational initiation	Eif4ebp2	insulin receptor signaling pathway	unknown	NM_010124
nuclear mRNA splicing, via spliceosome	Sf sc35	DNA binding	spliceosome complex	AF077858
nuclear mRNA splicing, via spliceosome	Sf 3b2	unknown	nucleus	BC049118
nuclear mRNA splicing, via spliceosome	Sf rs6	pre-mRNA splicing factor	nucleus	NM_026499
phospholipid biosynthesis	Gpam	acyltransferase	mitochondrion	NM_008149
positive regulation of transcription	Mafb	DNA binding	transcription factor complex	AF180338
post-embryonic development	Sepp1	selenium binding	extracellular space	NM_009155
protein biosynthesis	RPs21	structural constituent of ribosome	ribosome	BC027563
protein biosynthesis	RPs7	RNA binding	ribosome	NM_011300
protein folding	DnaJ	unfolded protein binding	membrane	NM_019794
protein ubiquitination	Zfp364	ubiquitin-protein ligase activity	ubiquitin ligase complex	NM_026406
proteolysis and peptidolysis	Cts e	neutrophil collagenase activity	extracellular space	NM_007799
proteolysis and peptidolysis	Cts h	cysteine-type endopeptidase activity	lysosome	NM_007801
proteolysis and peptidolysis	Cts l	cysteine-type endopeptidase activity	lysosome	NM_009984
proteolysis and peptidolysis	TMPRSS2	trypsin activity	integral to membrane	NM_015775
regulation of cell shape	Sprr 2A	constituent of cytoskeleton	cornified envelope	BC010818
regulation of muscle contraction	Cnn1	calmodulin binding	unknown	NM_009922
regulation of muscle contraction	Myl19	calcium ion binding	myosin	XM_283793
response to oxidative stress	Prdx1	antioxidant activity	unknown	NM_011034
response to oxidative stress	Prnp	copper ion binding	Golgi apparatus	NM_011170
synaptic transmission	Syn1	protein dimerization	synaptic vesicle membrane	NM_013680
tRNA aminoacylation	Lamp2	tRNA ligase	platelet dense granule membrane	NM_010685
unknown	RPCI23	unknown	unknown	AC078911
unknown	Mgs1-182e11	unknown	unknown	AC096622
unknown	RP23-70M6	unknown	unknown	AC114820
unknown	Tcra-V13.1	unknown	unknown	AE008684
unknown	Upk1a	unknown	integral to membrane	AF262335
unknown	SAC1	unknown	integral to membrane	AJ245720
unknown	Elp3	N-acetyltransferase activity	mitochondrion	AK012072
unknown	Ambladder	unknown	unknown	AK020558
unknown	MLZE	unknown	unknown	AK037079
unknown	Amcq	unknown	unknown	AK080832
unknown	RP23-452N23	unknown	unknown	AL928645
unknown	Endomuc1	unknown	integral to membrane	BC003706
unknown	UDP-gluco	unknown	unknown	BC019434
unknown	Sdfr1	receptor activity	integral to membrane	NM_009145
unknown	Serpina3n	endopeptidase inhibitor	extracellular space	NM_009252
unknown	Ddx3	ATP-dependent helicase activity	intracellular	NM_010028
unknown	Gus	unknown	unknown	NM_010368
unknown	Tnnt3	unknown	unknown	NM_011620
unknown	Ifrg15	unknown	unknown	NM_022329
unknown	2310015N07	unknown	unknown	NM_025515
unknown	Thsd6	unknown	extracellular space	NM_025629
unknown	Upk1b	unknown	integral to membrane	NM_178924
unknown	Aplp2	serine-type endopeptidase inhibitor activity	integral to membrane	U15571
unknown	Mpp1	protein binding	membrane	U38196
unknown	My h11	unknown	unknown	XM_147228
unknown	Lrrfip2	unknown	unknown	XM_284541

### SSH-selected transcripts

Table 3 summarizes the isolated transcripts that in general are involved in: actin dynamics (*wdr1*); apoptosis (*sirt1, siva*, and *bcap31*); calcium ion binding (*pls3, sc1, scp-1, sparc*, and *smoc2*); cell adhesion (*col3a1 *and *catenin*); cell cycle/ G-protein coupled receptor (*cam2*); cell growth (*prss11*); cell motility (*actr3*); cytoskeleton organization and biogenesis (*actg1*, *actg2*, and *mylc2b*);defense response (*ly6d*); electron transport (*cox7b*, *grp58*, and *coxI*); epithelial cell proliferation (*grn*); immune response (*ifit3*); insulin processing (*cpe*); iron ion transport (*fth1*); metabolism/ glutathione transferase activity (*gsto1*, *gsta4*, and *gstm1*); negative regulation of translational initiation (*eif4ebp2*); nuclear mRNA splicing (*sfsc35 *and *sfrs6*); phospholipid biosynthesis (*gpam*); positive regulation of transcription (*mafb*); post-embryonic development (*sepp1*); protein biosynthesis (*rps7 and rps21*); protein folding (*dnaJ*); protein ubiquitination (*zfp364*); proteolysis (*ctse*, *ctsh*, *ctsl*, *Aplp2*, *serpina3n*, and *tmprss2*); regulation of cell shape (*sprr2A*); regulation of muscle contraction (*my19 *and *cnn1*); response to oxidative stress (*prdx1 *and *prnp*); synaptic transmission (*syn1*); and tRNA aminoacylation (*lamp2*).

### Target validation by quantitative real-time polymerase chain reaction (QRT-PCR)

From the annotated transcripts (Table 2), twenty six were selected for further analysis by QRT-PCR. The results are summarized in Table 4. In tissues isolated from saline-treated mice the following transcripts were expressed preferentially in the detrusor muscle when compared to the mucosa layer: *ctsh*, *eif4ebp2*, *gstm1*, *gsto1*, *serpina3n*, *sprr2A*, *upk1a*, and *upk1b*. With the exception of *serpina3n*, all detrusor-specific transcripts were also preferentially up regulated in the inflamed detrusor. In contrast, *calm2, cnn1*, and *smoc2 *were preferentially expressed in the bladder mucosa of control mice. With the exception of *calm2*, all other mucosa-specific transcripts were up-regulated during inflammation. Table 4 also segregates transcripts that were represented in both layers of control mice and that were up-regulated during the inflammatory process. The latter include: *bcap31, catenin, pls3, mafb, prss11, mpp1, syn1, lamp2*, and *sepp1*.

**Table 4:** Differential expression of selected SSH transcripts by quantitative real-time polymerase chain reaction (QRT-PCR) ***

**Genes**	**Normalized CT values**								**log2 CT X1000000**				Fold Change (Delta CT Values)*				
										
	**Average (n = 3)**				**SEM**												
	
	**LD**	**LM**	**CD**	**CM**	**LD**	**LM**	**CD**	**CM**	**LD**	**LM**	**CD**	**CM**	**CD/CM**	**CM/CD**	**LD/LM**	**LD/CD**	**LM/CM**
Cts h	26.5	24.2	26.1	24.1	0.03	0.01	0.05	0.01	96	19	70	18	**3.9**	0.3	**5.0**	1.4	1.1
Eif4ebp2	36.5	30.4	33.7	30.9	0.62	0.07	0.07	0.05	95252	1430	13833	1974	**7.0**	0.1	**66.6**	**6.9**	0.7
Gst m1	23.4	19.1	23.2	18.2	0.32	0.02	0.00	0.06	11	1	9	0	**29.9**	0.0	**19.7**	1.2	1.8
Gst o1	36.0	26.9	35.5	29.8	0.14	0.17	0.56	0.06	68660	125	47023	909	**51.7**	0.0	**550.9**	1.5	0.1
Serpina3n	21.8	21.1	26.1	23.5	0.03	0.02	0.09	0.05	4	2	72	12	**6.1**	0.2	1.6	0.1	0.2
Sprr 2A	29.4	21.8	25.4	19.8	0.06	0.14	0.03	0.00	697	4	45	1	**51.0**	0.0	**185.1**	**15.3**	**4.2**
Upk1a	32.5	24.4	30.0	23.7	0.18	0.01	0.02	0.13	6162	23	1050	14	**76.4**	0.0	**271.4**	**5.9**	1.7
Upk1b	26.5	22.2	26.7	21.4	0.04	0.02	0.12	0.05	98	5	112	3	**39.3**	0.0	**20.6**	0.9	1.7
Calm2	23.2	24.0	22.2	23.8	0.11	0.09	0.04	0.11	9	17	5	14	0.3	**3.0**	0.6	2.0	1.2
Cnn1	32.1	33.1	27.2	31.0	0.50	0.09	0.15	0.05	4547	9400	149	2177	0.1	**14.6**	0.5	**30.5**	**4.3**
Smoc2	33.9	34.7	26.0	28.4	0.06	0.27	0.04	0.06	15953	27402	68	352	0.2	**5.2**	0.6	**234.8**	**77.7**
Bcap31	33.3	29.2	26.7	26.9	0.40	0.07	0.08	0.01	10331	627	106	127	0.8	1.2	**16.5**	**97.6**	**4.9**
Catenin	36.9	33.0	27.1	28.0	0.23	0.07	0.42	0.09	130085	8592	143	273	0.5	1.9	**15.1**	**912.8**	**31.5**
Mafb	32.1	30.1	31.8	30.5	0.65	0.10	0.34	0.44	4703	1154	3615	1570	2.3	0.4	**4.1**	1.3	0.7
Pls3	37.4	35.3	28.6	29.3	0.10	0.18	0.14	0.15	179012	41906	402	661	0.6	1.6	**4.3**	**444.9**	**63.4**
Prss11	29.9	27.9	25.8	24.5	0.05	0.03	0.06	0.00	999	259	59	23	2.5	0.4	**3.9**	**17.1**	**11.0**
Syn1	30.1	25.3	24.1	22.8	0.22	0.04	0.21	0.13	1148	42	18	7	2.4	0.4	**27.3**	**65.6**	**5.9**
Lamp2	31.6	31.1	28.1	27.8	0.21	0.14	0.05	0.01	3342	2379	285	230	1.2	0.8	1.4	**11.7**	**10.3**
Mpp1	31.0	30.0	29.2	29.5	0.44	0.11	0.11	0.19	2114	1090	632	753	0.8	1.2	1.9	**3.3**	1.4
Sepp1	23.0	23.9	22.0	21.6	0.06	0.03	0.08	0.05	8	15	4	3	1.4	0.7	0.6	2.0	**4.8**
Thsd6	28.1	28.3	28.3	28.0	0.13	0.08	0.03	0.04	293	329	338	272	1.2	0.8	0.9	0.9	1.2
IFIT3	30.4	31.2	30.8	30.9	0.08	0.00	0.12	0.05	1455	2396	1885	1988	0.9	1.1	0.6	0.8	1.2
Ifrg15	27.9	27.9	28.2	27.6	0.03	0.05	0.09	0.07	254	245	307	210	1.5	0.7	1.0	0.8	1.2
Sdfr1	24.4	25.6	24.1	24.8	0.06	0.09	0.10	0.09	23	52	18	29	0.6	1.6	0.4	1.3	1.8
Siva	26.7	25.8	27.1	25.8	0.03	0.07	0.06	0.07	110	59	147	59	2.5	0.4	1.9	0.7	1.0
Prnp	24.1	25.7	23.3	24.6	0.02	0.07	0.02	0.00	18	53	11	26	0.4	2.5	0.3	1.7	2.0

*(ratio of antilog2 of cycle threshold values)

** (bolded cells indicate values greater than 3.0)

***Female C57BL/6J mice were instilled with saline (n = 20) or LPS (n = 20). Twenty four hours after LPS instillation, mice were euthanized, the bladder was removed and placed in RNAlater™ (Ambion) for separation of the mucosa and submucosa from the detrusor smooth muscle, as described in Material and Methods. Four sample groups were obtained as follows: control mucosa (CM), control detrusor (CD), LPS-treated mucosa (LM), and LPS-treated detrusor (LD). The QRT-PCR amplifications were accomplished on an ABIPRISM 7700 using SYBRGreen I dye assay chemistry

All samples were run in triplicate with the appropriate single QRT-PCR controls (no reverse transcriptase and no template). From the QRT-PCR data, an average cycle threshold (Ct) value was calculated from the triplicate reactions. Averaged Ct values were then normalized (to adjust for different amounts of cDNA within each reaction) to the exongenous control gene, RCA. The relative expression level of each transcript within each sample group (CD, CM, LD, and LM) was determined by calculating the ratio of the antilog2 of the delta Ct values.

### TREs

Figure 2 contains over-represented TREs and the downstream transcripts that were found primarily in the bladder mucosa when compared to detrusor muscle isolated from control mice (MC). In contrast, figure 5 contains over-represented TFs and transcripts that were found primarily in the control detrusor muscle (DC). TFs and downstream transcripts specifically expressed in bladder mucosa during inflammation were compared to control mucosa (Figure 3) or to the inflamed detrusor (Figure 4). In contrast, the response of detrusor muscle to inflammation was determined in comparison to detrusor control (Figure 6) or inflamed mucosa (Figure 7). The results presented here demonstrate the concept that combining SSH methodology with PAINT-guided transcriptional regulatory element analysis permitted the generation of testable hypotheses regarding differences between mucosa and detrusor regulatory networks in health and disease states.

**Figure 1:**
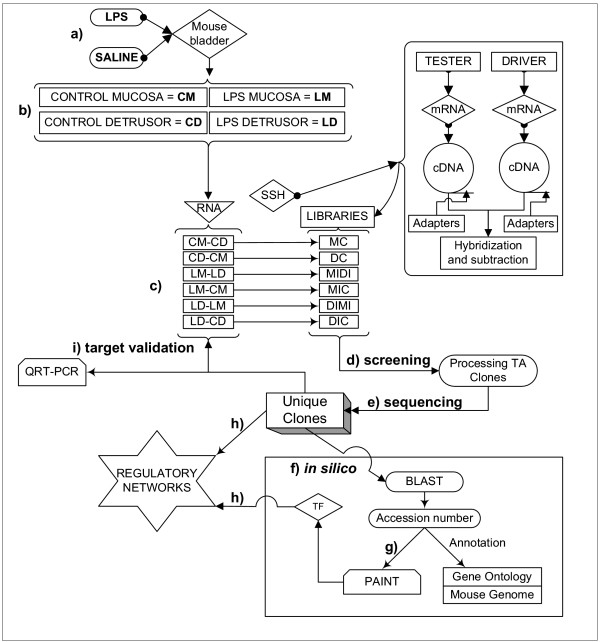
**Overall experimental design**. a) A well established animal model of LPS- induced bladder inflammation was used. b) RNA was extracted from isolated detrusor muscle and mucosa layers. c) Extracted RNA was used to generate 6 different libraries by suppressive subtraction hybridization (SSH) in order to determine bladder tissue- and treatment- dependent transcripts. d) Transcripts were then screened and e) Sequenced f) All unique transcripts were fully annotated by querying public (PubMed, Gene Ontology, Mouse Genome [108]) and private (Transfac professional [109]) databases. g) The accession number of each SSH-selected transcript was uploaded into the PAINT 3.3 feasnet builder [110] to query the Transfac database [109]. h) A regulatory network for each library was originated by a combination of SSH-selected transcripts and over-represented TF (0 < p < 0.05) in the matrix when compared to the PAINT database reference equivalent to the all the genes in the Ensembl annotated genome (Figures 2, 3, 4, 5, 6, 7). i) Unique clones were validated by QRT-PCR.

**Figure 2:**
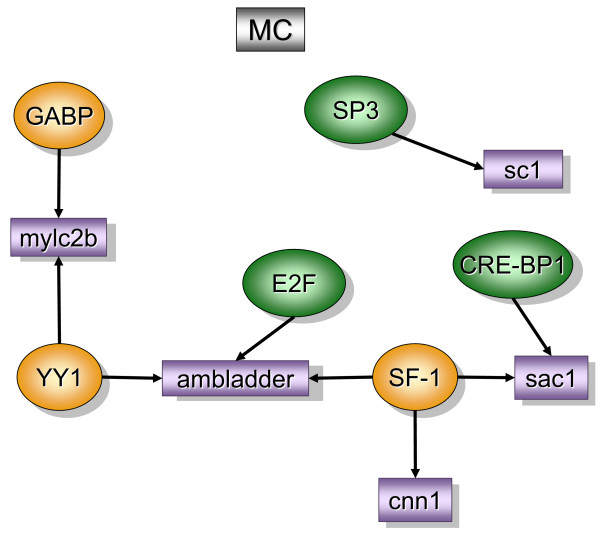
**Transcripts and TREs over-represented in the control mucosa when compared to control detrusor (MC)**. The regulatory network was determined by a combination of SSH-selected transcripts (green) and PAINT 3.3 query of transcription factor database (TRANSFAC). PAINT 3.3 was employed to examine 2000 base pairs of regulatory region upstream of the transcriptional start site of each differentially expressed transcript detected with the SSH. Genbank accession numbers were used as the gene identifiers in PAINT test files. Individual elements of the matrix are colored by the significance *p*-values. Over-representation in the matrix when compared to the reference (all TFs in the PAINT database) is indicated in orange (0 < p < = 0.01) or green (0.01 < p < 0.05). For a detailed origin of each library please see Figure 1 and Material and methods.

**Figure 3:**
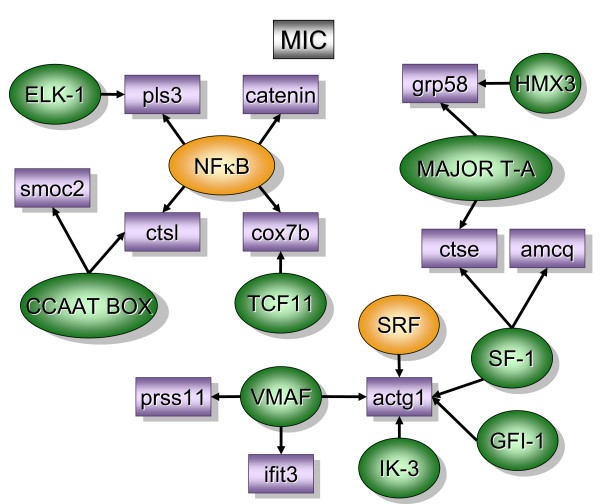
**Transcripts and TREs over-represented in the mucosa inflamed when compared to control mucosa (MIC)**. The regulatory network was determined by a combination of SSH-selected transcripts (green) and PAINT 3.3 query of transcription factor database (TRANSFAC). PAINT 3.3 was employed to examine 2000 base pairs of regulatory region upstream of the transcriptional start site of each differentially expressed transcript detected with the SSH. Genbank accession numbers were used as the gene identifiers in PAINT test files. Individual elements of the matrix are colored by the significance *p*-values. Over-representation in the matrix when compared to the reference (all TFs in the PAINT database) is indicated in orange (0 < p < = 0.01) or green (0.01 < p < 0.05). For a detailed origin of each library please see Figure 1 and Material and methods.

**Figure 4:**
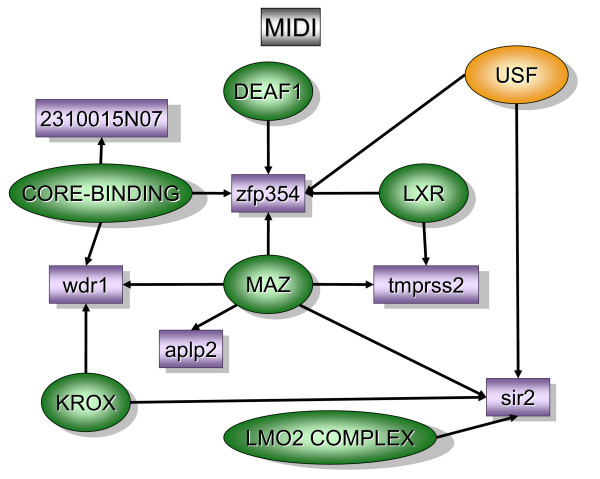
**Transcripts and TREs over-represented in the mucosa inflamed when compared to detrusor inflamed (MIDI)**. The regulatory network was determined by a combination of SSH-selected transcripts (green) and PAINT 3.3 query of transcription factor database (TRANSFAC). PAINT 3.3 was employed to examine 2000 base pairs of regulatory region upstream of the transcriptional start site of each differentially expressed transcript detected with the SSH. Genbank accession numbers were used as the gene identifiers in PAINT test files. Individual elements of the matrix are colored by the significance *p*-values. Over-representation in the matrix when compared to the reference (all TFs in the PAINT database) is indicated in orange (0 < p < = 0.01) or green (0.01<p < 0.05).

**Figure 5:**
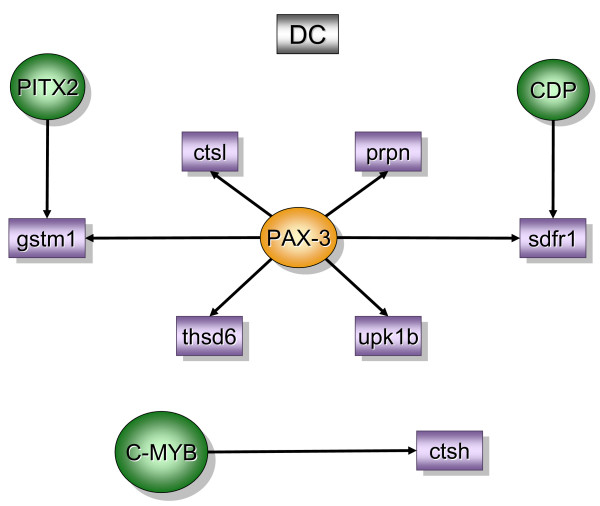
**Transcripts and TREs over-represented in the control detrusor when compared to control mucosa (DC)**. The regulatory network was determined by a combination of SSH-selected transcripts (green) and PAINT 3.3 query of transcription factor database (TRANSFAC). PAINT 3.3 was employed to examine 2000 base pairs of regulatory region upstream of the transcriptional start site of each differentially expressed transcript detected with the SSH. Genbank accession numbers were used as the gene identifiers in PAINT test files. Individual elements of the matrix are colored by the significance *p*-values. Over-representation in the matrix when compared to the reference (all TFs in the PAINT database) is indicated in orange (0 < p < = 0.01) or green (0.01 < p < 0.05). For a detailed origin of each library please see Figure 1 and Material and methods.

**Figure 6:**
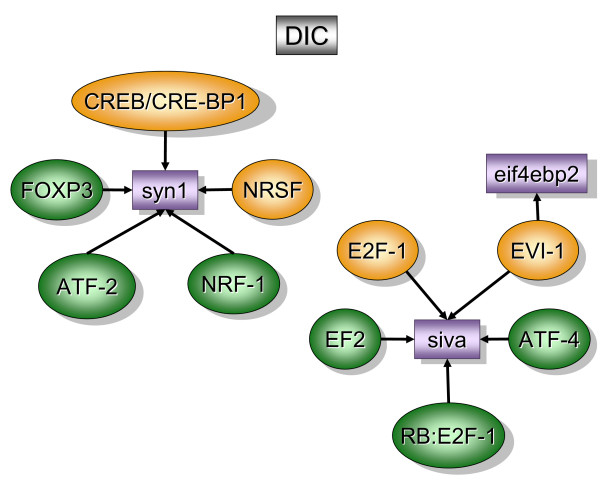
**Transcripts and TREs over-represented in the detrusor inflamed when compared to detrusor control (DIC)**. The regulatory network was determined by a combination of SSH-selected transcripts (green) and PAINT 3.3 query of transcription factor database (TRANSFAC). PAINT 3.3 was employed to examine 2000 base pairs of regulatory region upstream of the transcriptional start site of each differentially expressed transcript detected with the SSH. Genbank accession numbers were used as the gene identifiers in PAINT test files. Individual elements of the matrix are colored by the significance *p*-values. Over-representation in the matrix when compared to the reference (all TFs in the PAINT database) is indicated in orange (0 < p < = 0.01) or green (0.01 < p < 0.05). For a detailed origin of each library please see Figure 1 and Material and methods.

**Figure 7:**
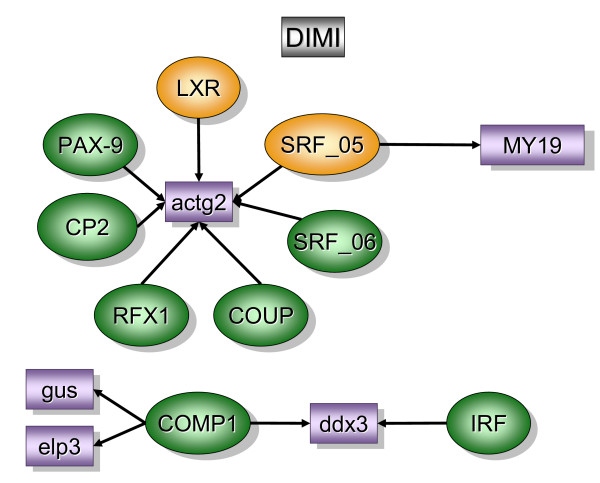
**Transcripts and TREs over-represented in the detrusor inflamed when compared to mucosa inflamed (DIMI)**. The regulatory network was determined by a combination of SSH-selected transcripts (green) and PAINT 3.3 query of transcription factor database (TRANSFAC). PAINT 3.3 was employed to examine 2000 base pairs of regulatory region upstream of the transcriptional start site of each differentially expressed transcript detected with the SSH. Genbank accession numbers were used as the gene identifiers in PAINT test files. Individual elements of the matrix are colored by the significance *p*-values. Over-representation in the matrix when compared to the reference (all TFs in the PAINT database) is indicated in orange (0 < p < = 0.01) or green (0.01 < p < 0.05). For a detailed origin of each library please see Figure 1 and Material and methods.

## Discussion

We used a highly effective method for differential gene analysis, termed suppression subtractive hybridization (SSH), which has been developed for the generation of subtracted cDNA libraries. It is based primarily on suppression PCR and combines normalization and subtraction in a single procedure [18]. The normalization step equalizes the abundance of cDNAs within the target population and the subtraction step excludes the common sequences between the target and driver populations. In a model system, the SSH technique enriched for rare sequences over 1,000-fold in one round of subtractive hybridization [18]. Unlike microarrays, which mainly identify moderate to high abundant genes, SSH identifies clones that are expressed at very low levels. It is possible that some of the extremely low-level gene expression is not biologically significant, as it might arise from 'random transcription' [19]. Therefore, we confirmed the differential expression of twenty six SSH-selected transcripts by *QRT-PCR *and the results were highly correlated. In addition, we introduced a redundancy factor by comparing the data generated from the analysis of multiple SSH libraries (MC, DC, MIC, MIDI, DID, and DIMI). Nevertheles, the question of validation certainly is an important one, and it is one we have considered. For one, we examined properties of the candidate genes that were independent of expression, such as the presence of promoter sequences, to increase the probability that mechanisms suggested by the expression data were not simply statistical anomalies. Moreover, the finding that mechanisms known to be operant emerged from the analysis also increases confidence that novel ones are valid as well.

This study was carried out at a single time point. The 24 hour time point was chosen because it coincides with the peak of acute inflammation [8,9]. This point characterizes the acute inflammatory responses to LPS and in addition to edema and vasodilation, the peak response of neutrophil infiltration occurred at 24 h in the mucosa and submucosa as well as in the detrusor smooth muscle [8,9]. The rationale for a single time point was to keep the number of variables within a reasonable limit. We understand that 24 h time point may not identify preceding or proceeding events as we indicated previously [7-9]. Therefore, the results of regulatory network here presented should be viewed as a snapshot of the inflammatory transcriptome at the point of maximal inflammatory response. Nevertheless, genes identified as important at this time point can now be followed over time by other techniques.

By using cDNA subtractions between mucosa and detrusor smooth muscle layers isolated from control and inflamed bladders, several clones identified transcripts that were further annotated. It has to be taken in consideration that the mucosal layer contains the urothelial layer and the lamina propria which involves other cell types (fibroblasts, myofibroblasts, etc.) in addition to urothelial cells that may underlie the transcriptomes identified. In addition, during the inflammation, inflammatory cells are present in the detrusor as well as in the mucosa and may contribute to the inflammatory bladder transcriptome.

Some of the transcripts are RIKEN sequences and therefore, have no biological process attributed. Interestingly, some of these transcripts with unknown function, such as *ambladder*, have been reported in the adult male urinary bladder by others using SSH [17]. However, the present work indicates that the same transcript was isolated from female urinary bladder as well.

Central to elucidation of hypothetical cis-regulatory networks is the identification and classification of naturally occurring transcription factor-binding sites in subtracted libraries. The combination of SSH-derived transcripts with PAINT-guided query of the TRANSFAC database permitted the generation of regulatory networks containing upstream TREs connecting corresponding TFs to the respective transcripts. Interestingly, many of the genes identified by SSH are transcription factors which emphasize the use of SSH to identify low expression transcripts.

### Mucosa control [MC], (figure 2)

The regulatory network for the control bladder mucosa (MC) was obtained in comparison to detrusor control and contained the following over-represented TREs: GA-binding protein (GABP), YY1 (yin yang 1), SF-1 (steroidogenic factor-1), EF2, CRE-BP1, and Sp-3 (trans-acting transcription factor 3).

GABP, also known as nuclear respiratory factor 2 (NRF-2), is a transcriptional coordinator of mitochondrial and nuclear-encoded subunits of cytochrome oxidase genes [20]. NRF-2 responds to increased neuronal activity by translocating from the cytoplasm to the nucleus, where it engages in transcriptional activation of target genes [21]. GABP is abundant in the kidney [22], however, the information about GABP is scanty in the rest lower urinary tract.

YY1 is a zinc finger TF which is thought to regulate cell growth and differentiation. YY1 normally antagonizes serum response factor (SRF) [23]. Interestingly, in the inflamed mucosa YY1 was not expressed and SRF was considered over-represented (Figure 2). YY1 along with GAPB drives the expression of myosin light chain phosphatase (*mylc2b*) which can be developmentally regulated in mammalian urinary bladders [24] and it is involved in the bladder response to obstruction [25].

SF-1 is a zinc finger motif of nuclear receptors [26] essential for steroidogenesis as well as for the development of the reproductive axis [27]. CRE-BP1, which is an ubiquitous basic-leucine zipper, is required for normal skeletal development.

YY1, SF-1, and EF2 were found upstream of *ambladder *(RIKEN clone 9530014P05 or prothymosin alpha). Prothymosin alpha is an oxidative stress-protecting gene [28] and transgenic mice over-expressing this transcript develop polycystic kidney disease PKD [29]. Another gene downstream of SF-1 was calponin 1 (*cnn1*). *Cnn1 *encodes for a multifunctional protein whose expression is tightly restricted to differentiated smooth muscle cell lineages during embryonic and post-natal life [30].

SF-1 and CRE-BP1 were found upstream of *sac1 *(a murine homolog of S. cerevisiae suppressor of actin mutations). Finally, an additional TRE, Sp-3 was found upstream of *sc1 *which is a calcium binging protein and an extracellular matrix protein precursor.

Epithelial-stromal interactions in bladder development have been extensively studied [5]. However, the present results depicted in the MC regulatory network raises the hypothesis that the bladder mucosa exhibits TREs and genes whose proteins may regulate the smooth muscle phenotype (Figure 2).

### Detrusor control [DC] (figure 5)

The regulatory network for the control detrusor smooth muscle suggests that Pax-3 plays a central role. In addition to Pax-3, other TREs such as: PITX2, CDP, and c-Myb were also found over-represented (figure 5).

Pax-3 was found upstream of the following transcripts: prion protein (*prnp*), cathepsin L (*ctsl*), stromal cell derived factor receptor 1 (*sdfr1*) [31], thrombospondin, type I domain containing 6 (*thsd6*), and uroplakin 1b (*upk1b*). *Prnp *seems to be involved in cell-to-cell interaction and its expression has been observed in germ cell differentiation during spermatogenesis [32]. *Ctsl *is a lysosomal proteolytic enzyme and an imbalance between *ctsl *and its inhibitors is believed to correlate with bladder tumor progression [33].

In addition to *ctsl*, the detrusor also presents *ctsh *under the control of c-Myb. Interestingly, *thsd *is involved in bladder cancer development [34] and is a target for methylation [35]. In addition, *thsd *has been described as inhibitor of angiogenesis. Finally, *upk1b *gene is highly expressed in normal human urothelium and its mRNA was undetectable or markedly reduced in bladder carcinoma [36].

Interestingly, the control detrusor normally expresses genes in the Pax-3 pathway that maintain neural progenitor cells [37] and myoblasts [38] undifferentiated. Therefore, our data suggests that Pax-3-regulated suppression of neural development in control detrusor changed substantially during inflammation and genes involved in neuronal development such as *syn *were found to be up-regulated. The later implies that detrusor instability may be a consequence of alterations in Pax-3 pathway leading to increased maturation of neural progenitor cells within the bladder smooth muscle.

### Mucosa inflamed versus control [MIC]. (figure 3)

Of all regulatory networks (Figures 2, 3, 4, 5, 6, 7), MIC was the only one presenting NF-κB (p < 0.01), (Figure 3). These results confirmed our previous observation that it is the bladder mucosa and in particular, the urothelium, that responds to LPS with NF-κB translocation [39]. This independent identification of NF-κB [39], which is known to play a key role in bladder inflammation [40] strengthens confidence in the identification of novel pathways.

Three transcripts were found downstream of NF-κB: *catenin *(*c*adherin-associated protein, delta 1), *cox7b*, and *pIs3 *(plastin 3 T-isoform). Regarding *catenin*, others have described its presence in the bladder mucosa and in particular in the urothelium [41]. Indeed, *alpha1-catenin *was reported to be reduced in bladder urothelial cells treated with anti-proliferative factor [42] which make this transcript a possible target in cystitis. Moreover, p120-catenin is frequently altered and/or lost in tumors of bladder [43]. Finally, bladder cancers harboring a *beta-catenin *mutation may represent aggressive biological behavior with enhanced proliferating activity [44]. *Cox7b *encodes cytochrome C oxidase, subunit VIIb which is the terminal component of the mitochondrial respiratory chain and catalyzes the electron transfer from reduced cytochrome C to oxygen. Finally, *pls3 *was found by differential display to have increased expression in cisplatin-resistant human cancer cells [45].

In addition to NF-κB, MIC also had SRF that along with several zinc finger TFs (SF-1 [Steroidogenic factor 1]; Gfi-1 (growth factor independent 1); ik-3 (Ikaros 3); and vMaf [basic region leucine zipper]), drive the activity of *actg1. Actg1 *is a highly conserved protein involved in various types of cell motility, and maintenance of the cytoskeleton.

The MIC regulatory network also includes the following unique transcripts: *smoc2 *(secreted modular calcium-binding protein 2), *amcq *(adult male corpora quadrigemina cDNA; RIKEN clone:B230340L02), *prss11 *(Serine protease 11), and *ifit3 *(Interferon-induced protein with tetratricopeptide repeats 3). *Smoc2 *is a widespread glycoprotein with a calcium-dependent conformation [46]. *Prss11 *encodes a secreted trypsin (HtrA1) that regulates the availability of insulin-like growth factors (IGFs) by cleaving IGF-binding proteins and therefore, may function as a regulator of cell growth [47]. HtrA is involved in stress response pathways [48]. Interestingly, down-regulation of *prss11 *expression may be an indicator of melanoma progression [49].

*Ifit3 *was originally cloned from a cDNA library prepared from the murine cell line, RAW 264.7, after bacterial LPS stimulation [50]. Although it is a transcript induced by IFN [51], its function is still unknown.

*Grp58 *encodes a ubiquitously expressed chaperone protein that resides in the endoplasmic reticulum and is part of the protein folding machinery [52]. It is likely that *grp58 *is involved in the oncogenic transformation[53] since expression analysis revealed an up-regulation of *grp58 *in breast, uterus, lung, and stomach tumors [54].

Together these results confirmed that the bladder mucosa expresses unique transcripts involved in cell growth, motility, and cytoskeleton, protein folding, and proteases. Some of these transcripts have been described to be altered in cystitis as well as in LPS-induced bladder inflammation. However, the most striking result was that of 6 different networks, the MIC library was the only one to have over-representation of NF-κB.

### Mucosa inflamed versus detrusor inflamed [MIDI], (figure 4)

The question being answered by this experiment was whether or not the bladder mucosa sets the stage for LPS-induced inflammatory responses by up-regulating a unique set of genes and TFs distinct from the detrusor muscle.

In terms of TFs, the upstream stimulatory factor (USF) was the most significantly over-represented (p < 0.001) and driving unique transcripts (figure 4). USF dimerizes to regulate transcription through E-box motifs in target genes. Although widely expressed, they can mediate tissue-specific transcripts. USF is stimulated by glucose in murine mesangial cells, binds to TGF-β1 promoter, contributes to TGF-β1 expression, and may play a role in diabetes-related gene regulation in the kidney [55]. Others have shown that USF binding activity is enhanced in response to LPS [56].

Another interesting TF was the liver X-activated receptors (LXR) found upstream of several transcripts bearing USF sequence. LXR is a member of the nuclear receptor superfamily [57] is a negative regulator of macrophage inflammatory gene expression [58], and a putative therapeutic agent for the treatment of inflammation [58], diabetes [59], and neurodegenerative diseases [57,58,60].

LIM-only proteins (LMO), which consist of LMO1, LMO2, LMO3, and LMO4, are involved in cell fate determination and differentiation during embryonic development [61]. LMO2 was originally identified through its involvement in T-cell leukemia and subsequently shown to be critical for normal hematopoietic and endothelial development [61]. Accumulating evidence suggests that LMO1 and LMO2 act as oncogenic proteins in T-cell acute lymphoblastic leukemia, whereas LMO4 has recently been implicated in the genesis of breast cancer[61].

MAZ (Myc-associated zinc finger protein), also known as serum amyloid A-activating transcription factor-1 (SAF-1), plays a major role in regulating transcription of several inflammation-responsive genes, including matrix metalloproteinase-1 (81), the mouse mast cell protease (mMCP)-6 [62], and function as growth suppressor in fibroblasts [63]. SAF-1 transgenic mice are prone to develop a severe form of inflammation-induced arthritis [64]. MAZ is upstream of Aplp2 which is a key regulator of structure and function of developing neuromuscular synapses [65].

In terms of unique transcripts isolated from the MIDI library, our results indicate that transcripts such as *zfp364 *(zinc finger protein 364, also known as *rab7*) and *sir2 *are downstream of series of TFs including: USF, LXR, DEAF1, MAZ, and Lmo2 complex (figure 4). *Sir2 *gene encodes a member of the sirtuin family of proteins which are NAD-dependent histone/protein deacetylases [66]. *Zfp364 *is a member of the Rab family of small G proteins, and regulates intracellular vesicle traffic to late endosomes [67]. Another unique transcript was the androgen-regulated *tmprss2 *protease [68] known to be expressed in urogenital tissues [69]. *Tmprss2 *has gained interest owing to its highly localized expression in the prostate and its over-expression in neoplastic prostate epithelium. Once activated, the serine protease domain of *tmprss2 *is released from the cell surface into the extracellular space and activates PAR (protease-activated receptor)-2 that has a role in prostate cancer and tumor metastasis [70]. Among the proteins correlating with cytoskeleton dynamics, our SSH identified a transcript (*wdr1*) encoding a 67-kDa WD40 repeat protein 1 which is the vertebrate homologue of actin-interacting protein 1 [71]. *Wdr1 *is involved in actin dynamics and seems to be required to induce cell morphologic changes, especially mitotic cell rounding [72]. Others have shown that *wdr1 *was found upregulated in the lung [73] and cell lines [74] following exposure to nickel oxide-induced carinogenesis. Finally, MIDI library also contained a RIKEN cDNA 2310015N07 gene that was described to be isolated from developing mouse libraries but no function yet has been attributed [75].

Interestingly, a comparison between MIDI and DIMI libraries indicates that both share LXR as a TRE. The major difference between these two libraries was found downstream LXR activation. In the inflamed mucosa, LXR preferentially activates *zfp364 *and *tmprrs2 *whereas in the inflamed detrusor LXR was found as a co-modulator of *actg2*.

In conclusion, the mucosa regulatory network presents USF in a central position raising the hypothesis that USF-target promoters such as the TGF-β1 promoter are involved in the mucosal response to inflammation and whether mucosa inflammation follows similar diabetes- related mucosal gene expression.

### Detrusor inflamed versus detrusor control [DIC], (figure 6)

The regulatory network of the detrusor muscle, inflamed versus control, selected the following transcripts: *syn1 *(Synapsin I or ribosomal protein S15a), *siva *(CD27-binding protein), and *eif4ebp2 *(negative regulation of translational initiation), (figure 6).

*Syn1 *is a member of the synapsin gene family which is a neuron-specific phosphoprotein of small synaptic vesicles.*Syn1 *has been mapped to an evolutionarily conserved linkage group composed of: *araf1*, *syn1*, *timp*, and *properdin *located at human chromosome Xp11.2 [76] and mouse chromosome X [77]. Of interest, *araf1 *is a proto-oncogene which is predominantly expressed in mouse urogenital tissues [77]. In contrast, *siva *has an important role in the apoptotic pathway induced by the CD27 antigen. Others have described that *siva *is a direct transcriptional target for both tumor suppressors, p53 and E2F1 [78]. Finally, the eukaryotic initiation factor *eIF4E *and eIF4E-binding proteins (4E-BPs) control the initiation of protein synthesis and are part of a translational signaling pathway sensitive to insulin [79] and rapamycin [80]. Changes in the state of phosphorylation of *eIF4E *and 4E-BPs occur at an early stage of apoptosis [81]. Interestingly, eIF4E selectively enhances the translation of powerful angiogenic factors such as FGF-2 and VEGF [82] and therefore may have a role in oncogenesis [82] as well as inflammation.

Over represented TFs in DIC regulatory network were NRSF (Kruppel-type zinc-finger transcriptional repressor RE1-silencing transcription factor [REST]; also known as the neuron-restrictive silencing factor), CREB/CRE-BP1, E2F-1, and Evi-1.

CREB/CRE-BP1, also called transcription factor ATF-2, binds to the cAMP response element and its activity is enhanced after phosphorylation by stress-activated protein kinases such as c-Jun N-terminal kinase and p38. ATF-2 plays a central role in TGFβ signaling by acting as a common nuclear target of both Smad and TAK1 pathways [83].

Nrf-1 (nuclear respiratory factor 1) regulates expression of nuclear-encoded mitochondrial genes and it was shown to be part of the response to LPS in rats [84].

FOXp3 belongs to the forkhead gene family which comprises a diverse group of "winged-helix" TFs with important roles in development, metabolism, cancer and aging [85]. Recently, several forkhead genes have been demonstrated to play critical roles in lymphocyte development and effector function [85]. FoxP3 is a potential target for treatment of experimental chronic inflammatory renal disease [86] and type I diabetes [87]. In addition, both FOXp3 and NRSF seems to be downstream of Wnt-Frizzled signaling [88] which was recently proposed to participate in the pathogenesis of interstitial cystitis [89].

E2F, E2F-1, and Rb-E2F-1 belong to a family of TFs implicated in the regulation of cell proliferation and their binding sites are present in the promoters of several growth-regulating genes. E2F family members are functionally regulated, in part, by complex formation with one or more members of the nuclear pocket protein family such as the retinoblastoma protein (Rb) and play a role in neuronal development [90] by acting as negative regulator of cell proliferation. The interplay between Rb and E2F is critical for proper cell cycle progression [91]. Of interest, E2F-1 has a growth-promoting effect in bladder superficial TCC [92].

ATF-3 (activating transcription factor 3) is transcriptional repressor involved in survival and regeneration of sensory neurons [93,94] that responds to insulin [95]. ATF3 is also a novel stress-activated regulator of p53 protein stability/function providing the cell with a means of responding to a wide range of environmental insults [96]. In addition, ATF3 represents a novel mechanism in which anti-inflammatory drugs exert their anti-invasive activity [97].

The proposed role of *nrsf/rest *is that of a transcriptional silencer that restricts neuronal gene expression to the nervous system by silencing their expression in non-neural tissues [98]. Interestingly, loss of *nrst *function in human prostate carcinoma cells is associated with neuroendocrine phenotype, tumor progression, and androgen independence [99]. Others investigators indicated that *nrst *also modulates the cholinergic gene locus [100,101] which may have some implication in detrusor instability. Recently, it was proposed that activation of the *rest/nrsf *target genes overrides muscle differentiation pathways and converted myoblasts to a physiologically active neuronal phenotype [102]. It remains to be determined whether *nrst *promotes the same transformation in the inflamed detrusor muscle. The latter would explain the hyperactivity of detrusor muscle observed in over-active bladder disorders such as obstruction, incontinence, and inflammation.

In conclusion two major networks are proposed to be active in the detrusor inflamed when compared to control. One containing a neuron-specific phosphoprotein of small synaptic vesicles (*syn*) and the other an important protein of apoptotic pathway (*siva*). In both cases, analysis of the intense upstream promoter network leads us to the hypothesis that both genes represent a common downstream target of several pro-inflammatory stimuli.

### Detrusor inflamed versus mucosa inflamed [DIMI], (figure 7)

The question being answered by this experiment was whether or not the inflamed detrusor muscle expresses unique transcripts and TFs distinct from the bladder mucosa.

Two major pathways could be constructed with the combination of SSH and PAINT results. The first involves key smooth muscle proteins, a myosin light chain encoded by *my19 *and gamma actin encoded by *actg2*. Gamma actins are highly conserved proteins that are involved in various types of cell motility, and maintenance of the cytoskeleton. In addition, a role for smooth muscle alpha actin in force generation by the urinary bladder has been suggested [103]. Several TREs upstream of *actg 2 *and *my19 *were over-represented in MIDI, including LXR which was described above, SRF, COUP, Pax-9, CP2, and RFX1.

A second pathway involved the transcripts *elp3, gus, and ddx3 *and two TFs COMP1 and IRF. *Ddx3 *is a putative RNA helicase and a member of a highly conserved DEAD box subclass. RNA helicases are highly conserved enzymes involved in transcription, splicing, and translation [104]. There are several examples of the involvement of RNA helicases in differentiation of germ cells, particularly in spermatogenesis. Upstream of *ddx3*, PAINT selected a TF that cooperates with myogenic proteins (COMP1) and Interferon Regulatory Factor (IRF). Transcription of IRF is synergistically activated by products of inflammation such as IFNγ and TNFα [105].

Two other transcripts were found downstream of COMP1: *Gus *(beta-glucoronidase) and *elp3*. Gus is a sensitive indicator LPS activation of macrophages. *Elp3 *is one of the sub units of the elongator complex, an acetyltransferase important for normal histone acetylation involved in elongation of RNA polymerase II transcription.

By comparing the inflamed detrusor and mucosa (DIMI), the fundamental difference observed was the up-regulation in the detrusor of genes and TFs related to smooth muscle function. It is fair to propose that this network could underlie detrusor instability during inflammation.

## Conclusion

We here present a novel approach to understanding the bladder response to inflammation as a system. By using SSH, low abundance, differentially expressed transcripts could be detected that probably would have been lost in the background "noise" of a microarray study. That these genes were, in fact, key players was shown by the remarkable concordance in the transcriptional regulatory elements identified and by target validation with *QRT-PCR*. We suggest that the results identified key players governing the normal growth and differentiation of bladder mucosa and urothelium as well as the cross-communication of these layers during inflammation resulting from a number of pathologic processes.

As genes encoding DNA-binding TFs are the largest class of genes involved in human oncogenesis, it was obvious that in several instances the vast amount of information was related to cancer, in general and to bladder carcinoma, in particular. Interestingly, some of the TFs and their correlated downstream transcripts originally described to be involved in organogenesis were also activated during inflammation. The implications of these findings may represent one more link between inflammation and cancer.

The networks here described could well represent key targets for development of novel drugs for treatment of bladder diseases.

## Methods

### Animals

Ten to twelve-week old female C57BL/6J mice were used in these experiments that were performed in conformity with the "Guiding Principles for Research Involving Animals and Human Beings (OUHSC Animal Care & Use Committee protocol #002-109).

### Induction of inflammation

Acute inflammation was induced by instillation of LPS into the mouse bladder, as described previously [8,9,106]. Female mice were anesthetized (ketamine 200 mg/kg and xylazine 2.5 mg/kg, i.p.), then transurethrally catheterized (24 Ga.; 3/4 in; Angiocath, Becton Dickson, Sandy, Utah), and the urine was drained by applying slight digital pressure to the lower abdomen. Because the bladder of 10-week old mice has an average capacity of 250 μl, the urinary bladders were instilled with 200 μl of one of the following substances: pyrogen-free saline (control) or *Escherichia coli *LPS strain 055:B5 (Sigma, St. Louis, MO; 100 μg/ml),) (figure 1a). Substances were infused at a slow rate to avoid trauma and vesicoureteral reflux. To ensure consistent contact of substances with the bladder, infusion was repeated twice within a 1-hour interval and a 1-ml syringe was maintained in the catheter end during this period. The catheter was removed, and mice were allowed to void normally. Twenty-four hours after instillation, mice were euthanized with pentobarbital (100 mg/kg, i.p.) and bladders were removed rapidly.

### Tissue layers – separating the mucosa from detrusor

Immediately after removal from the animal, bladders were placed in RNA*later*™ (Ambion) and visualized under a dissecting microscope (Nikon SMZ 1500). The detrusor smooth muscle was separated by blunt dissection away from the mucosa which contained the epithelium and sub-epithelial layers, (see Additional file 1).

### Suppression subtractive hybridization (SSH) (9)

A total of six libraries were obtained by SSH (figure 1c). In order to standardize the names of the groups and to correlate with delta CT values (see below), the SSH libraries were named after the *tester *minus *driver*. The first library (MC) was obtained by using the mucosa removed from control saline-treated mice (CM) as *tester *and the respective detrusor smooth muscle (CD) as *driver*. The resultant subtraction MC was supposed to contain genes preferentially expressed in the control mucosa. The second library was the reverse of MC and therefore, CD was used as *tester *and CM as *driver *and will contain genes preferentially expressed in the detrusor smooth muscle of control mice (DC). The other 4 libraries were obtained to investigate genes whose expression was altered during LPS-induced inflammation (figure 1c). The samples used for each SSH were obtained by pooling RNA from 20 individual mice. The pooling was necessary in order to obtain enough RNA from each layer without amplification.

### Construction of subtractive cDNA libraries

mRNA was isolated from total RNA using Poly(A) Quick mRNA Isolation Kit (Stratagene, La Jolla, CA) according to the manufacture's protocol. To compare the two populations of resulting cDNA the method of SSH was performed using PCR-Select cDNA Subtraction Kit (BD Biosciences – Clontech, Palo Alto, CA), as described by Diatchenko and colleagues [18]. This method selectively amplifies differentially expressed sequences, and the generation of high- and low-abundance sequences is equalized during the first hybridization. The PCR allows amplification of equalized differentially expressed sequences. Each step of the cDNA synthesis and subtractive hybridization procedure was monitored using the positive control samples provided by the manufacturer. We verified the efficiency of subtraction by PCR analysis by comparing GAPDH levels in subtracted and un-subtracted cDNA using the method and GAPDH primers provided by the manufacturer. For analysis of efficiency, please see Additional file 2. For analysis of ligation, please see Additional file 3. For the analysis of PCR products, please see Additional file 4. For PCR analysis and subtraction efficiency, please see Additional file 5.

Next, cDNAs from the testers and drivers were digested with *Rsa*I. To select tissue- and treatment-specific transcripts, PCR adapters were ligated to the tester pool population. The tester cDNA pool was then hybridized with excess cDNAs from the driver pool. After hybridization suppression, PCR using primers specific for the tester PCR adapters selectively amplified differentially expressed transcripts.

### Screening the clones (plating out, growing up and analyzing the PCR clones), (figure 1d)

After the PCR subtraction, the amplification products were cloned into the pCR 2.1 plasmid of the TA cloning kit (Invitrogen). Ligated DNA was transformed by heat shock in 100 μl of INVαF competent *E. coli *cells. Colonies were grown overnight at 37°C on Luria broth agar plates containing ampicillin, X-gal, and isopropyl-B-D-thiogalactopyranoside for blue/white colony selection. White colonies were isolated and grown individually in 2 ml of LB medium containing ampicillin for 16 h. After plasmid DNA isolation (Wizard^® ^*Plus *SV Minipreps DNA Purification System – Promega), digestion was performed using *Xba *I and *Bam*H I, and the products analyzed in 1% agarose gels. Positive clones (representing fragments larger than the original polylinker in the cloning vector) were sent to a sequencing service (figure 1e), and sequences were submitted for a BLAST analysis in GenBank for identification and annotation was done by searching the gene ontology[16], figure 1f.

### Tissue- and treatment-specific transcription factors

After annotation, a bioinformatics approach to identify functionally relevant putative transcriptional regulatory elements (TREs) for all SSH-selected transcripts was used (figure 1g). We used the Promoter Analysis and Interaction Network Toolset [PAINT [13]], available online [107], to integrate functional genomics information from SSH-derived gene expression data with the genomic sequence and TRE data to derive hypotheses about relevant transcriptional regulatory networks. PAINT uses the TRANSFAC^® ^database [14] of transcription factors and position weight matrix descriptions of cis-acting sequences and an associated pattern matching tool MATCH [15] to identify statistically over-represented regulatory sites in 5' upstream sequences of related genes. This information provides a substantially pruned list of TFs regulating tissue- and treatment-specific genes that were identified by SSH. Briefly, the accession numbers of all the SSH-selected transcripts were used as an input gene list in PAINT. Up to 2000 base pairs of 5' upstream sequences were analyzed for the presence of TREs using a MATCH/TRANSFAC setting to minimize false positives and filtering the results further in PAINT to consider only those hits with 100% match to the 5 bp core TRE sequence. The interaction matrix contained 79 genes and 162 TREs.

PAINT can analyze the interaction matrix for over-represented TREs in subsets/clusters of related genes, typically grouped based on gene expression data. The library for each transcript (MC, DC, MI, MIDI, DI and DIMI) was considered as the cluster label and provided as the Gene-Cluster information in the PAINT analysis and visualization step. The TRE over-representation in PAINT is calculated as the hyper-geometric probability of the observed number of TREs in a given cluster as compared to that in randomly selected gene clusters from a 'reference' list. In this study, all the genes in the genome as annotated in Ensembl were used to construct the interaction matrix for use as the reference. To construct the hypothesized regulatory networks, TFs were chosen based on the probability of over-representation (p < 0.05) in any of the six gene groups as compared to all the genes in the genome (or equivalently in PAINT promoter database).

### Target validation by QRT-PCR (figure 1i)

#### RNA isolation and cDNA synthesis

An additional 40 C57BL/6J female mice, ten to twelve-weeks old underwent the same intravesical treatment as described above [LPS, (n = 20 mice) and saline (n = 20 mice)]. Twenty four hours after LPS instillation, mice were euthanized, the bladder was removed and placed in RNA*later*™ (Ambion) for separation of the mucosa and submucosa from the detrusor smooth muscle, as described above. Four sample groups were obtained as follows: control mucosa (CM), control detrusor (CD), LPS-treated mucosa (LM), and LPS-treated detrusor (LD). Bladders were pooled and homogenized in Ultraspec RNA solution (Biotecx Laboratories, Houston, TX) for isolation and purification of total RNA used for QRT-PCR. High RNA quality from each of these four groups was verified by capillary gel electrophoresis using an Agilent 2100 Bioanalyzer (Agilent Technologies, Inc, Palo Alto, CA). RNA concentration was determined by spectrophotometry using a NanoDrop^® ^ND-1000 UV-Vis Spectrophotometer (Nano Drop Technologies, Wilmington, DE). Subsequently, this total RNA was used as a template for each of the first-strand cDNA syntheses. Prior to cDNA synthesis, an exogenous standard of *A. thaliana *(RCA) mRNA (Stratagene, L Jolla, CA, USA) was added (0.1 ng) to each CM, CD, LM, and LD total RNA (2 μg) sample for normalization of succeeding gene expression data. RNA was reverse-transcribed according to the Omniscript RT™ kit (Qiagen, Valencia, CA) instructions and subsequently purified using the Montage PCR 96-well cleanup plate (Millipore, Billerica, MA). Prior to the PCR, primer pairs were designed utilizing both Primer Express^® ^(ABI, Foster City, CA) and NetPrimer (PREMIER Biosoft International, Palo Alto, CA) software. Primers were designed according to the general guidelines outlined in the Primer Express^® ^User Bulletin. Details of the primers and the Genebank accession numbers are given in Table 1. The designed primers shared 100% homology with the target sequence but no significant homology with other sequences.

The QRT-PCR amplifications were accomplished on an ABI^®^PRISM 7700 using SYBR^®^Green I dye assay chemistry. A 15 μL PCR assay for each gene of interest consisted of 7.5 μL of 2X SYBR^®^Green PCR mix (Applied Biosystems Inc., Foster City, CA), 4.9 μl of H20, 0.6 μl (30 pmoles) of gene-specific forward and reverse primers, and 2 μl (1 ng) of cDNA template. All samples were run in triplicate with the appropriate single QRT-PCR controls (no reverse transcriptase and no template). Cycling conditions used for all amplifications were one cycle of 95°C for 10 minutes and 40 cycles of 95°C for 15 seconds and 60°C for 1 minute. Following the QRT-PCR, dissociation curve analysis was performed to confirm the desired single gene product.

From the QRT-PCR data, an average cycle threshold (Ct) value was calculated from the triplicate reactions. Averaged Ct values were then normalized (to adjust for different amounts of cDNA within each reaction) to the exongenous control gene, RCA. The relative expression level of each transcript within each sample group (CD, CM, LD, and LM) was determined by calculating the ratio of the antilog_2 _of the delta Ct values. The resultant fold-change data is presented in Table 4.

## Authors' contributions

**MRS **participated in its design, carried out the animal experiments, removed the tissues, performed suppression subtractive hybridizations, and performed sequence alignments. **NBN **helped MRS on the suppression subtractive hybridizations experiments. **HLH **participated in the design of the study and trained both MRS and NBN to develop SSHs. **MT **and **MC **developed Q-PCR.**RV **developed the PAINT program and guided **RS **to perform TF analysis. **DWD's **laboratory sequenced all transcripts. **REH **participated in its design and helped to draft the manuscript. **RS **conceived of the study, developed annotation and TF analysis, and draft the manuscript.

## Supplementary Material

Additional File 1 Separation of bladder layers (mucosa and detrusor). Click here for file

Additional File 2 Detailed methodology for SSH and analysis of the experimental procedures. Click here for file

Additional File 3 Analysis of ligation. Click here for file

Additional File 4 Analysis of PCR products. Click here for file

Additional File 5 The reduced message of a known housekeeping gene between the subtracted and un-subtracted cDNA over the same number of PCR cycles. Click here for file
