# Supplementary material for: The inflammatory and normal transcriptome of mouse bladder detrusor and mucosa
Source: BMC Physiol. 2006 Jan 18;6:1. doi: 10.1186/1472-6793-6-1 (PMC1382248; doi:10.1186/1472-6793-6-1)
Supplement: Additional File 1 — Separation of bladder layers (mucosa and detrusor). [file 1472-6793-6-1-S1.pdf]

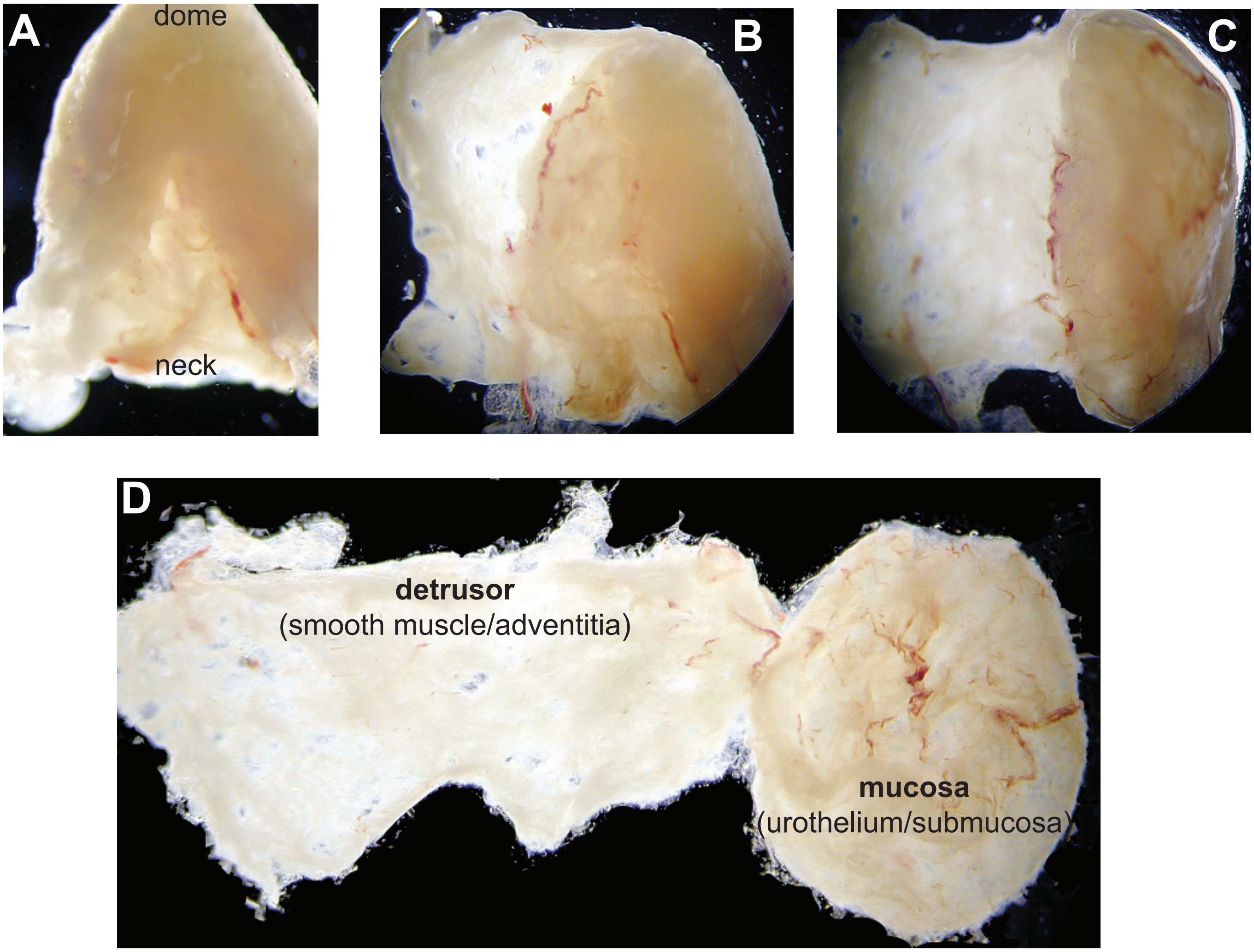

Additional file 1 A-D.

Separation of bladder layers (mucosa and detrusor). Immediately after removal from the animal, bladders were placed in RNAlater™ (Ambion) and visualized under a dissecting scope (Nikon SMZ 1500). The detrusor smooth muscle and adventitia was separated by blunt dissection away from the mucosa containing the urothelium and sub-epithelial layers (**1 A-D**). An incision was made on the bladder neck and a forceps introduced between the detrusor and the mucosa. Careful dissection of the layers indicated a clear demarcation of a white detrusor apart from a yellowish mucosa (Additional files **1-B** and **1-C**). In addition, blood vessels further helped to identify the two layers. The end result was the isolation of two layers of tissue, one containing the whole epithelium and submucosa and the other containing the detrusor muscle and adventitia (**1-D**). In separated experiments, cross-sections of the dissected layers indicated that the method here employed lead to a minimum degree of cross-contamination between detrusor and mucosa (data not shown). Whole tissues were photographed at room temperature by a Nikon digital camera (DXM1200; Nikon, Japan). Images were imported into Adobe Photoshop 9.0, and then placed on Adobe InDesign CS2 in which groups of images were assembled and labeled. Composite figures were printed on Adobe Acrobat 7.0 (Adobe Systems, San Jose, CA).
