# Supplementary material for: The inflammatory and normal transcriptome of mouse bladder detrusor and mucosa
Source: BMC Physiol. 2006 Jan 18;6:1. doi: 10.1186/1472-6793-6-1 (PMC1382248; doi:10.1186/1472-6793-6-1)
Supplement: Additional File 2 — Detailed methodology for SSH and analysis of the experimental procedures. [file 1472-6793-6-1-S2.pdf]

**Detailed Methodology for SSH.**

There were six steps in this procedure: I – Double stranded cDNAs were synthesized from tester and driver mRNAs and digested with RsaI to generate optimal fragments for hybridization reactions. II – Adapters Ad1 and Ad2R were ligated to two separate populations of the tester cDNAs. III –Tester cDNAs were then, mixed with 30X excess driver cDNAs. The mixtures were processed in the first hybridization to normalize and enrich differentially expressed sequences among single strand (ss) tester molecules. IV – The reactions from the first hybridization were mixed and processed for a second hybridization in the presence of additional driver ss cDNAs, resulting in combination of different hybrid types. V – The ends of the respective hybrids were filled in to generate cDNA fragments from differentially expressed genes that can be preferentially amplified by PCR using appropriate primers. VI - Two rounds of PCR were performed to preferentially amplify deferentially expressed genes.

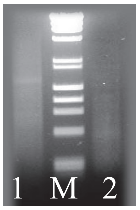


Additional File 2

**Additional file 2. Analysis of the Experimental procedures.** The efficiency of cDNA synthesis and RsaI digestion was verified by agarose gel electrophoresis. **Additional file 2** shows typical result of cDNA synthesis (Lane 1) and after (Lane 2) RsaI digestion. Lane M: DNA size markers.
