# Supplementary material for: The inflammatory and normal transcriptome of mouse bladder detrusor and mucosa
Source: BMC Physiol. 2006 Jan 18;6:1. doi: 10.1186/1472-6793-6-1 (PMC1382248; doi:10.1186/1472-6793-6-1)
Supplement: Additional File 3 — Analysis of ligation. [file 1472-6793-6-1-S3.pdf]

**Additional file 3. Analysis of Ligation.** Following PCR experiment, we verify that at least 25% of the tester cDNAs have adaptors on both ends. A typical result demonstrating the ligation efficiency is shown in **additional file 3**. Lane 1 is the forward subtraction PCR with adaptor primers and Lane 2 is the PCR with the genes specific primers. Lanes 3 and 4 are the reverse subtraction ligation efficiency. Lane 3 is PCR with adaptor primers, and Lane 4 is PCR with gene specific primers. M: DNA size primers.

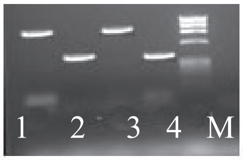


Additional File 3
