# Supplementary material for: The inflammatory and normal transcriptome of mouse bladder detrusor and mucosa
Source: BMC Physiol. 2006 Jan 18;6:1. doi: 10.1186/1472-6793-6-1 (PMC1382248; doi:10.1186/1472-6793-6-1)
Supplement: Additional File 4 — Analysis of PCR products. [file 1472-6793-6-1-S4.pdf]

Additional file 4. Analysis of PCR Products. This step is to verify is enough rounds of PCR amplification were performed. Comparing the subtracted (Lane 1) and un-subtracted (Lane 2) PCR products, typical results are presented in **additional file 4**. The banding pattern from the subtracted to un-subtracted cDNA should be different for a successful subtraction. PCR analysis of Subtraction Efficiency. The efficiency of forward subtraction was evaluated by PCR. This was done by comparing the abundance of known cDNAs before and after subtraction.

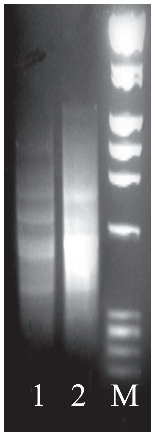

Additional File 4
