# Supplementary material for: The inflammatory and normal transcriptome of mouse bladder detrusor and mucosa
Source: BMC Physiol. 2006 Jan 18;6:1. doi: 10.1186/1472-6793-6-1 (PMC1382248; doi:10.1186/1472-6793-6-1)
Supplement: Additional File 5 — The reduced message of a known housekeeping gene between the subtracted and un-subtracted cDNA over the same number of PCR cycles. [file 1472-6793-6-1-S5.pdf]

Additional file 5 shows the reduced message of a known housekeeping gene between the subtracted and un-subtracted cDNA over the same number of PCR cycles.

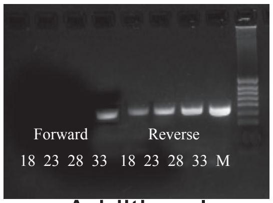

Additional File 5
